# Induced Neurons for Disease Modeling and Repair: A Focus on Non-fibroblastic Cell Sources in Direct Reprogramming

**DOI:** 10.3389/fbioe.2021.658498

**Published:** 2021-03-12

**Authors:** Kathryn M. Kim, Mentor Thaqi, Daniel A. Peterson, Robert A. Marr

**Affiliations:** ^1^Center for Neurodegenerative Disease and Therapeutics, Chicago Medical School, Rosalind Franklin University of Medicine and Science, North Chicago, IL, United States; ^2^Scholl College of Podiatric Medicine, Rosalind Franklin University of Medicine and Science, North Chicago, IL, United States

**Keywords:** direct reprogramming, induced neurons, brain repair, neurogenesis, glia

## Abstract

Direct cellular reprogramming exhibits distinct advantages over reprogramming from an induced pluripotent stem cell intermediate. These include a reduced risk of tumorigenesis and the likely preservation of epigenetic data. *In vitro* direct reprogramming approaches primarily aim to model the pathophysiological development of neurological disease and identify therapeutic targets, while *in vivo* direct reprogramming aims to develop treatments for various neurological disorders, including cerebral injury and cancer. In both approaches, there is progress toward developing increased control of subtype-specific production of induced neurons. A majority of research primarily utilizes fibroblasts as the donor cells. However, there are a variety of other somatic cell types that have demonstrated the potential for reprogramming into induced neurons. This review highlights studies that utilize non-fibroblastic cell sources for reprogramming, such as astrocytes, olfactory ensheathing cells, peripheral blood cells, Müller glia, and more. We will examine benefits and obstructions for translation into therapeutics or disease modeling, as well as efficiency of the conversion. A summary of donor cells, induced neuron types, and methods of induction is also provided.

## Introduction

Neurons are the primary functional unit of the brain and are a diverse, dynamic, and essential cell population of great importance to our study of cognitive function as well as therapeutic development for brain injury. Direct reprogramming of somatic cells to various subtypes of induced neurons (iN) has great potential in the field of neuroscience research. This approach allows for disease modeling in neurons of human origin (even from the same patient) as well as the possibility for developing regenerative medicine therapies. The interest in developing direct reprogramming techniques can be attributed to advantages that arise from technical differences between induced pluripotent stem cell (iPSC)-derived iN and directly reprogrammed iN derived from somatic cells. The direct reprogramming approach can utilize endogenous patient cells as donors, removing the need for the complicated and expensive process of iPSC derivation, reprogramming, and engraftment. Furthermore, it reduces the risk of tumorigenesis/teratogenesis due to the lack of a pluripotent intermediate state and holds the potential of preserving the epigenetic memory of the donor cell, which has a tremendous impact on the accuracy of disease modeling. Using an epigenetic biomarker of aging, DNA methylation, data showed that epigenetic age of the donor cell was consistent with the corresponding directly reprogrammed iN (Huh et al., 2016). It is reported that, in iPSC-derived neurons, the age-related epigenetic information is, for the most part, lost during the deprogramming process into the iPSC state, while direct reprogramming retains aging-associated gene expression changes (Mertens et al., 2015).

Direct reprogramming techniques can be grouped into two main categories, *in vitro* and *in vivo*. *In vitro* direct reprogramming methods have developed with the goal of more precise modeling of the pathophysiological development of disease and mapping potential therapeutic targets, while the *in vivo* direct reprogramming approach aims to treat various neurological disorders and injuries, by targeting the patient’s local endogenous cells for reprogramming (Srivastava and DeWitt, 2016; Fang et al., 2018). The common objective of both categories is the development of methods leading to better control of the subtype-specific iN. The majority of research has been centered on fibroblasts as the donor cell, partly as a result of the relative ease of obtaining and culturing fibroblasts. Here, we will highlight recent studies that utilize non-fibroblastic cell sources (Table 1). We will focus on the possible benefits and obstructions for translation into therapeutics or disease modeling and examine the efficiency and integrity of iN conversion.

**TABLE 1:** Overview of direct reprogramming techniques for non-fibroblastic cell sources.

Donor cell	Cell(s) produced	Method	References
Primary cortical astrocytes	iN, iNPC	Ngn2, CEND1 via retroviral vector (RV)	Aravantinou-Fatorou and Thomaidou, 2020
Region-restricted astrocytes	iN	Ngn2 via LV	Hu et al., 2019
Midbrain astrocytes	Noradrenergic iN	Ascl1, Phox2b, AP-2a, Gata3, Hand2, Nurr1, Phox2a via LV	Li et al., 2019
Astrocytes	Dopaminergic iN	NeuroD1, Ascl1, Lmx1a, miR218 (NeAL218) via LV	di Val Cervo et al., 2017
Astrocytes	iNPC→ further subtype specialization	Foxg1, Sox2, Brn2 with Lhx8 or Fox2a/Lmx1a via RV	Ma et al., 2018
Astrocytes	iN	NeuroD1 via adeno-associated virus (AAV) vector	Livingston et al., 2020
Parenchymal astrocytes	iN	Ascl1, Brn2a, Myt11 via LV	Torper et al., 2013
Dorsal midbrain astrocytes	iN	Ascl1 via AAV vector	Liu et al., 2015
Spinal astrocytes	Doublecortin-positive neuroblasts	Sox2 via LV	Su et al., 2014
Astrocytes	iN	NeuroD1 via AAV vector	Zhang et al., 2020
Astrocytes	iN	Ascl1, Prdx2, Sod1 via RV	Russo et al., 2020
Fetal cerebral cortex astrocytes	iN	Ascl1, LDN193189 and SB431542, DAPT, and VPA via protein transduction domain delivery	Robinson et al., 2018
Endogenous astrocyte	Nigral iN	AAV-vector mediated knockdown of PTB via shRNA	Qian et al., 2020
Adult brain pericytes	iN	Ascl1, Sox2 via RV	Karow et al., 2012, 2018
Human brain vascular pericytes	Cholinergic iN	Ascl1, Myt1l, Brn2, Tlx3, and miR124 via LV	Liang et al., 2018
Olfactory ensheathing cell	GABAergic iN, glutamatergic iN	Ngn2 via LV	Sun et al., 2019
Human urine cells	Glutamatergic iN	Small-molecule cocktail (CAYTFVB)	Xu et al., 2019
Human urine cells	iNSC	Small-molecule cocktail with self-replicable mRNA delivery	Kang et al., 2019
Adult peripheral blood mononuclear cells	Excitatory iN	Brn2, Ascl1, Myt1l, and Ngn2 via episomal vector	Tanabe et al., 2018
T lymphocytes	Excitatory iN	Brn2, Ascl1, Myt1l, and Ngn2 via episomal vector	Tanabe et al., 2018
Adult peripheral blood cells	iNPC? sensory iN	OCT4 with SMAD + GSK-3 inhibition via LV	Vojnits et al., 2019
CD 133-positive cord blood cells	iN	Sox2, c-Myc via Sendai virus	Castaño et al., 2014
Peripheral blood mononuclear cells	iN	Sox2, c-Myc via Sendai virus	Castaño et al., 2014
Peripheral blood mononuclear cells	Induced neural plate border stem cells	Brn2, KLF4, oxX2, and ZIC3 with four small molecule inhibitors via LV	Thier et al., 2019
Bone marrow-derived cells	iNPC	Msi1, Ngn2, MBD2 via non-integrating synthetic plasmid	Ahlfors et al., 2019
Cord blood CD133-positive cells	iNSC	Sox2, MYC, FOXM1, STAT6, and SALL4	Omrani et al., 2018
Adult peripheral CD34-positive cells	iN	AR, SMAD3, JUN, MYC, WT1, TAL1, SPI1, RUNX1, and Sox2	Omrani et al., 2018
Müller glia	iN	Ascl1, histone deacetylase inhibitor TSA, STAT inhibitor	Jorstad et al., 2020
Müller glia	Retina ganglion cells	Ngn2 or Ascl1 via plasmid	Guimarães et al., 2018
Müller glia	Neural retina progenitor cells	Bmi1, Spi1, Lmo2, Cebpd	Omrani et al., 2018
Müller glia	iN	Ascl1, histone deacetylase inhibitor TSA	Jorstad et al., 2017
Müller glia	Retinal ganglion cells, dopaminergic iN	CasRx-mediated Ptbp1 downregulation	Zhou et al., 2020
NG2 glia	GABAergic iN, glutamatergic iN	NeuroD1 via RV	Guo et al., 2014
NG2 glia in cerebral cortex	Doublecortin-positive iN	Sox2, Ascl1, or Sox2 via RV	Heinrich et al., 2014
NG2 glia in striatum	GABAergic iN, glutamatergic iN	Ascl1, Lmx1a, and Nurr1 via AAV	Torper et al., 2015
NG2 glia in striatum	GABAergic iN, glutamatergic iN	Ascl1, Lmx1a, Nurr1 via AAV5	Pereira et al., 2017, 2019
Glial progenitors (NG2 glia)	Midbrain dopaminergic iN	Ascl1, Lmx1a, Nurr1, shRNA to REST via LV	Nolbrant et al., 2020
Microglia	iN	NeuroD1 via LV	Trudler and Lipton, 2019
Hepatocyte cells	iN	Suz12, Ezh2, Meis1, Sry, Smarca4, Esr1, Pparg, and Stat3	Omrani et al., 2018
Glioma cells	Neuron-like cells	Ascl1 via LV	Cheng et al., 2019

## Characterization of Neurons, Reproducibility, and Integrity

The development of concrete criteria for the characterization of native neurons in comparison to iN-like cells is essential for effective translational therapeutic research. The extent to which an iN resembles an actual neuron plays an important role in modeling the disease process, thus providing more accurate pathophysiology and response to treatments. Identification of phenotypic markers consistent with neuronal lineage commitment and progression to maturity are important data to establish neuronal authenticity, but these markers alone cannot demonstrate functional neuronal maturation. A protocol developed to assess iN authenticity utilizes calcium imaging and electrophysiology as markers of conversion success (Hansen et al., 2019). Additionally, documentation of integration of iN into neural networking circuitry is strong evidence for successful reprogramming (Grønning Hansen et al., 2020).

The mechanistic approach of direct reprogramming is the forced expression of genes essential to eliciting a desired cellular identity leading to a rapidly induced fate conversion as compared to normal development. However, the corresponding changes in metabolism can cause an increase in reactive oxygen species that negatively impacts the health and survival of the iN (Gascón et al., 2016; Suzuki and Shults, 2019). This may have unforeseen consequences for the integrity of iN in a research model. The amount of evidence for transformation from a somatic cell to an iN and the effects of cell stress during the process of direct reprogramming are important factors for determining the viability for further applications.

## Fibroblasts

Direct reprogramming of fibroblasts has been well studied over the years and proven successful in producing a variety of subtype-specific neurons. Many direct reprogramming techniques using fibroblasts have been described in detail in other reviews (Traxler et al., 2019; Cates et al., 2021; Herdy et al., 2021). The use of other cell types is important because of the advantages they possess to better reprogram into and model the desired cellular subtype as well as the need to target various resident cell types in the target tissue.

## Astrocytes

Astrocytes are a promising target for *in vivo* applications of direct reprogramming for several reasons. Researchers have theorized that locality and lineage of the donor cell play a role in the efficacy of the direct reprogramming process. By targeting astrocytes for neural reprogramming, there would be no need for transplantation of cells from other locations in the body, potentially leading to fewer rejections and lowering risk of the formation of teratomas and other tumors. Astrocytes also proliferate and take part in glial scar formation in areas of injury and so are plentiful in regions requiring neuronal regeneration. Direct reprogramming of astrocytes using *in situ* mechanisms has the potential to pioneer a new alternative for regenerative medicine (Aravantinou-Fatorou and Thomaidou, 2020).

However, there are major obstacles in astrocyte reprogramming that require mitigation. The high frequency of cell death around the time of fate conversion is a significant limitation in reprogramming for astrocytes. Accordingly, ferroptosis inhibitors, antioxidants, and Bcl-2 led to improvements in conversion efficiency (Gascón et al., 2016). Additionally, researchers argued that methods that undergo an intermediate stage reduce oxidative stress and promote a safer reprogramming process (Gascón et al., 2016). One study reported that CRISPRa (activating)-mediated early induction of mitochondrial proteins improved metabolic conversion and reprogramming efficiency (Russo et al., 2020).

Research focused on gaining insight into the effects of astrocyte regional specificity reported that expression of Ascl1 via a GFAP promoter in an AAV vector in astrocytes located in the dorsal midbrain, striatum, and somatosensory cortex of both postnatal and adult mice led to reprogramming into iN (Liu et al., 2015). However, a recent study demonstrated the differential reprogramming capability of astrocytes in gray matter versus white matter when subjected to AAV mediated NeuroD1 transcription factor expression (Liu et al., 2020). Also, a more general study indicated that non-neuronal cells like astrocytes respond differently to the same transcription factor and result in iN with distinct molecular phenotypes depending on the brain region (Grande et al., 2013). Another study exhibited differences in conversion success depending on regional sources and destinations. Cortical astrocytes reprogrammed with Neurogenin-2 (Ngn2) into pyramidal-like neurons survived the transplantation process, while cerebellar astrocytes did not. However, astrocytes from both locations contributed iNs to the olfactory bulb following transplantation in the subventricular zone (Chouchane et al., 2017).

One study attributed limitations in conversion efficiency of astrocytes to their composite heterogeneity as compared to fibroblasts (Hu et al., 2019). This is corroborated in another study, which found that with similar methods, astrocyte conversion lagged behind fibroblasts by over 30% (Li et al., 2019). When a transcriptomic analysis compared noradrenergic iN derived from midbrain astrocytes versus mouse embryonic fibroblasts, they displayed significant similarity (Li et al., 2019). Despite their similar transcriptome, different signaling pathways were activated in murine embryonic fibroblast-derived iN which may indicate greater maturity than astrocyte-derived iN (Li et al., 2019). While fibroblast-derived iN may display greater maturity at baseline, another study indicated that astrocyte-derived induced neural progenitor cells (iNPC) displayed higher neurogenic competence than fibroblast-derived iNPC on whole transcriptome analysis (Xia et al., 2020). The astrocyte-derived iNPC had higher efficiency, mobility, and long-term survival, had stronger expression of Sox2, and were able to differentiate into specialized subtypes of forebrain neurons (Xia et al., 2020). Further studies are needed to elaborate on quantifiable differences in fibroblast-derived versus astrocyte-derived iN.

Induced neurons derived from astrocytes have been used for modeling and developing treatments for various neurodegenerative conditions, including Alzheimer’s disease, stroke, and Parkinson’s disease. Investigations include successful transplantation of astrocyte-derived noradrenergic iN into mouse brains, with evidence of integration into neural circuitry (Li et al., 2019). In another model, there was evidence for correction of gait impairments in Parkinson disease mouse models that underwent *in vivo* direct reprogramming with Cre-inducible lentiviral vectors (LV) for resident striatal astrocytes to tyrosine hydroxylase-expressing iN (Xia et al., 2020).

Direct reprogramming methods that manipulate the astrocyte gliosis response are being targeted for neuroregeneration (Aravantinou-Fatorou and Thomaidou, 2020). Reactive astrocytes in the cortex of stab-injured or Alzheimer disease model mice were directly reprogrammed into iN (Guo et al., 2014). Spontaneous and evoked synaptic responses in these glia-derived NeuroD1-converted neurons suggest that the iN integrated into local neural circuits (Guo et al., 2014). Increased plasticity in reactive astrocytes resulted in higher conversion rates when compared against non-reactive astrocytes (Brulet et al., 2017; Robinson et al., 2018). In a stroke model, *in vivo* direct reprogramming of astrocytes to iN following stroke resulted in improvements in motor function as soon as 3 weeks after the reprogramming process, with sustained long-term functional recovery (Livingston et al., 2020). Furthermore, cortical ischemia modeling has been performed, exploring direct reprogramming gene therapy for functional brain repair (Chen et al., 2019). In the spinal cord, *in vivo* conversion of astrocytes to neurons after acute injury resulted in successful neurogenesis (Su et al., 2014). A severe stab injury mouse model reported that NeuroD1-mediated reprogramming of reactive astrocytes into iN and reversed glial scar formation (Zhang et al., 2020).

While there has been evidence of success, some studies have reinforced the need for more effective administration strategies. One study reported low efficacy of direct reprogramming of astrocytes to iN post stroke in aged mice (Gresita et al., 2019). Researchers hypothesized that the therapeutic vectors carrying the conversion gene were phagocytosed rapidly by microglia after administration (Gresita et al., 2019). One noteworthy method used protein transduction domains fused to transcription factors to deliver proneural transcription factors to astrocytes (Robinson et al., 2018). This method lowers the risk of oncogenesis which is possible with some viral deliveries.

## Pericytes

A few recent studies have explored the use of pericytes, which play a critical role in formation of the blood–brain barrier, in direct reprogramming. Human brain vascular pericytes have multipotent stem cell potential, which provides an ideal cell context for lineage reprogramming (Liang et al., 2018). In one study, a combination of Ascl1 and Sox2 drove direct reprogramming of adult human vascular brain pericytes which resulted in bifurcation into distinct lineages (Karow et al., 2018). Analysis by scRNA-seq platforms showed that these lineages expressed specific inhibitory or excitatory neuronal transcription factors (Karow et al., 2018). A different approach to pericyte direct reprogramming reported that a single transcription factor, Myt1l, can successfully reprogram pericytes into cholinergic neuronal cells (Liang et al., 2018). BrdU-labeled experiments also showed that pericyte conversion led to cell cycle exit, which suggests that the iN did not pass through a pluripotent or proliferative state during the reprogramming process (Liang et al., 2018). This approach may reduce the risk of tumorigenicity for *in vivo* therapy research.

However, similar to astrocytes, pericyte heterogeneity greatly affects reprogramming competence. One possible explanation proposed is that there are at least two distinct populations of pericytes with divergent embryonic origins (Karow et al., 2018). In fact, the study may have underestimated the variability of pericytes, since they used retrovirus transduction which only involves transduction of relatively rapidly dividing cells (Karow et al., 2018).

## Olfactory Ensheathing Cells

Olfactory ensheathing cells have been noted for their ability to facilitate axonal regeneration and have been used in experiments assessing *in vivo* axonal repair (Li et al., 2003, 2005; Gu et al., 2019). Thus, it is possible that these cells would be a good target for direct reprogramming into neurons. Olfactory ensheathing cells share common molecular, morphological, and functional characteristics with astrocytes. They can be harvested in the olfactory mucosa, which provides easier access than astrocytes for sample collection (Sun et al., 2019). Olfactory ensheathing cells have a reported conversion rate of 80% and have an added benefit of displaying neuroprotective characteristics in non-reprogrammed olfactory ensheathing cells (Sun et al., 2019). While there is limited research available, so far, it has proved to be a valuable area of exploration.

## Urine-Derived Cells

Only a few recent studies have employed human urine cells in direct reprogramming. These studies have focused on transgene-free small-molecule transformation methods. Human urine cells are easily obtained at low risk and low cost without invasive procedures, only requiring urine collection in a sterile container. Another advantage is the reported rapid conversion in both studies mentioned, with complete reprogramming occurring at around 1 week (Kang et al., 2019; Xu et al., 2019). In the study that explored reprogramming of human urine cells to induced neural stem cells (iNSC), the resultant cells were able to undergo further differentiation to neurons, astrocytes, and oligodendrocytes (Kang et al., 2019). As with astrocytes, cell death from oxidative stress is a recurring problem in direct reprogramming as the somatic cell undergoes a metabolic transition. Human urine cell-derived iN had improved rates of survival with addition of Vitamin C (Xu et al., 2019). This method may be transferable to other direct reprogramming cell sources.

## Hematopoietic and Other Blood Cells

The use of blood cells in direct reprogramming is beneficial because it involves less invasive collection methods, as well as high availability for samples. In future studies, blood cells could be harvested from low-risk and healthy individuals as comparison tools against samples collected from individuals carrying the disease (Tanabe et al., 2018). Additionally, researchers found no significant change in efficiency between fresh or frozen blood cells, removing the need for fresh samples (Tanabe et al., 2018). For these reasons, blood-derived neurons have the capacity for large-scale modeling at lower cost. However, T cell-derived iN displayed less mature synaptic properties compared with iPSC-derived neurons (Tanabe et al., 2018). Further work is needed to improve on synaptic properties and maturation of induced cells. Initial studies showed that sensory iN derived from blood cells were able to model the effects of chemotherapy-related peripheral neuropathy (Vojnits et al., 2019).

## Müller Glia

Much of retinal Müller glial research is focused on understanding the differences in regenerative mechanisms between mammals and fish Müller glia (Ueki et al., 2015). Fish Müller glia exhibit retinal regeneration after injury, unlike mammalian Müller glia. Several studies have sought out methods for restoration of vision in retinal injured patients. One study reported a novel use of an RNA-targeting CRISPR system called CasRx that downregulates Ptbp1 and reprograms Müller glia into retinal ganglion cells (Zhou et al., 2020).

## Oligodendrocyte Precursor Cells

Oligodendrocyte precursor cells (OPC), or NG2 glia, are a promising donor cell source because of their abundance and proliferative capacity (Heinrich et al., 2014). NG2 glia can maintain their numbers after depletion making them an excellent target for regenerative medicine because cells lost to reprogramming could be replaced. Recent studies have shown that the use of Ascl1 alone did not induce any neurogenesis; however, NeuroD1 or Sox2 alone is sufficient to reprogram into iN (Guo et al., 2014; Heinrich et al., 2014). Recently, human glial progenitor cells (NG2 glial, fetal, or stem cell derived) were able to be reprogrammed into midbrain dopaminergic neurons by the expression of transcription factors Ascl1, Lmx1a, and Nr4a2 (Nurr1) along with inhibition of the RNA against the RE1-silencing transcription factor (REST) complex (Nolbrant et al., 2020).

## Other Cell Types

A recent study demonstrated the reprogramming potential of microglia into iN with NeuroD1 via LV at relatively high efficiency (25–35%) (Trudler and Lipton, 2019). Direct reprogramming is additionally being used in a new direction, glioblastoma cancer research. Researchers used Ascl1 to directly reprogram human glioma cancer cells to terminally differentiated neuron-like cells (Cheng et al., 2019). This novel approach to cancer treatment may allow clinicians to target tumors that are inoperable due to location or spread and are resistant to traditional chemotherapeutics targeting rapidly proliferating cells.

## Discussion

Direct reprogramming has tremendous potential. In one study, directly reprogrammed neural precursor cells administered during the subacute phase of stroke demonstrated promising results for recovery in motor skills (Vonderwalde et al., 2020). Another study reported significant improvement in motor function post transplant of iNSC into a mouse model of MS (Sullivan et al., 2020). Additional studies will be needed to better understand the extent to which addition of reprogrammed neurons can adequately modify a circuit to restore a functional output. Unambiguous cell identity remains an uncertainty within the direct reprogramming field, in particular determining when a cell has transformed into a neural-like cell versus a true neuron. There is a need for further transcriptomic analysis and a better mechanistic understanding of the reprogramming sequence. Epigenetic changes that indicate a neural identity are not always achieved with high fidelity (Trudler and Lipton, 2019). Furthermore, phenotypic and morphologic characteristics are not alone sufficient criteria and require demonstration of neuronal function. The need for functional analysis presents a major roadblock to the advancement of direct reprogramming applications in human patients. Related to this, one must consider the long-term *in vivo* survival of these cells, neural integration of iN into the appropriate circuitry, functional analysis at the cellular and network level, and finally the assessment of potential deleterious effects of iN introduction. Addressing these issues will help drive the field forward. With correlation of electrophysiology, calcium imaging, and integration into neural circuitry, preclinical studies have made significant strides toward producing a fully realized, directly reprogrammed iN.

## Author Contributions

KK completed a comprehensive review of the literature and produced initial drafts of the manuscript. MT provided draft review, edits, and corrections. DP provided content review and edits. RM supervised the activities of KK, produced edits and correction of drafts of the manuscript, and completed the final version of the text. All authors contributed to the article and approved the submitted version.

## Conflict of Interest

The authors declare that the research was conducted in the absence of any commercial or financial relationships that could be construed as a potential conflict of interest.
